# A nomogram based on clinical factors for preoperative prediction of nipple involvement in breast cancer

**DOI:** 10.3389/fsurg.2022.923554

**Published:** 2022-08-11

**Authors:** Weiling Huang, Zhikun Qiu, Tai Mu, Xi Li

**Affiliations:** ^1^ Department of Thyroid and Breast Surgery, The Third Affiliated Hospital of Sun Yat-sen University, Guangzhou, China; ^2^ Department of Breast Surgery, State Key Laboratory of Oncology in Southern China, Collaborative Innovation Center for Cancer Medicine, Sun Yat-sen University Cancer Center, Guangzhou, China; ^3^ Department of Breast Surgery, Huizhou Central People's Hospital, Huizhou, China; ^4^ Department of Thyroid and Breast Surgery, The First People's Hospital of Kashgar, Xinjiang, China; ^5^ Department of Surgery, Nyingchi People's Hospital, Nyingchi, China

**Keywords:** nipple-sparing mastectomy, nipple involvement, predictive, breast cancer, clinicopathologic characteristics

## Abstract

**Background:**

At present, the indication for nipple-sparing mastectomy (NSM) remains inconclusive, and occult nipple involvement (NI) is one of the most important problems when carrying out NSM. Therefore, we aimed to identify the predictive factors of NI, to provide a tool for selecting suitable candidates for NSM.

**Methods:**

In this retrospective study, a total of 250 breast cancer patients who received mastectomy were recruited, and the association between NI and tumor clinicopathologic characteristics was investigated. Nipple signs, tumor size measured by ultrasound (US), and tumor location were developed as a nomogram to predict NI.

**Results:**

Among the 250 patients, 34 (12.6%) had NI, and 216 (86.4%) did not. In the training group, NI was associated with nipple signs, tumor size, tumor–nipple distance (TND), tumor location, lymph node metastasis, and HER2 overexpression. Both in the training and in the validation groups, NI showed a significant association with nipple signs, tumor size measured by ultrasound, and tumor location. Based on these three clinical factors, the preoperative model nomogram was proved to have high efficiency in predicting NI, possessing a sensitivity of 80.0% and a specificity of 86.7% in the validation group.

**Conclusions:**

We proposed a predictive model nomogram utilizing preoperative tumor characteristics, including nipple signs, tumor size measured by ultrasound, and tumor location. This predictive model could help in the planning of nipple-sparing mastectomy.

## Background

Breast cancer is one of the three most common carcinomas in the world and endangers both the physical and the psychological health of women. With the advancement of locoregional and systemic therapy, the prognosis of breast cancer has substantially improved, and today, more attention is being paid to the quality of life in breast cancer patients (1, 2). Currently, breast reconstruction has become a standard of care owing to the positive influence on patients’ psychological health and social adaptation (3). Nipple-sparing mastectomy (NSM) is an increasingly used surgical approach that removes the whole breast tissue and the skin overlying superficial tumors, while preserving the nipple, which permits immediate breast reconstruction and effectively improves the cosmetic outcome of breast cancer surgery (4–6). As the nipple is an indispensable part of the breast, NSM provides much higher psychological satisfaction and improves patients' quality of life (7). Published studies have revealed a low incidence of local cancer recurrence after NSM in selected patients (8–13). Nevertheless, the preservation of the nipple remains a matter of concern due to occult nipple involvement (NI) (14). Thus, it is critical to develop clinical models to accurately predict occult nipple involvement for carrying out NSM effectively and safely. Previous studies have revealed a correlation between the rates of nipple involvement and clinicopathologic characteristics such as tumor size, tumor location, and tumor–nipple distance (TND), lymph node status, histological type, and nuclear grade of the tumor (15–20). Some models were also developed based on the clinicopathologic characteristics and MRI examination. However, the long scan time and relatively high costs limit the widespread use of MRI (21). In this study, we aimed to find the predictors of NI and established an inexpensive and easily available predictive model for surgical planning.

## Methods

### Study population

A total of 250 female patients diagnosed with breast carcinoma who had undergone mastectomy between May 2016 and June 2018 at the Third Affiliated Hospital of Sun Yat-sen University were included. Exclusion criteria consisted of the following: (1) lack of US examinations performed within 1 month before surgery; (2) missing pathological results of nipple status; and (3) incomplete records of physical examinations. All enrolled patients were randomly divided into training and validation cohorts in a ratio of 4:1.

### Clinicopathologic characteristics

The following information of patients was extracted from the case management system of our hospital. (1) Age. (2) Clinical nipple signs were deemed abnormal if there was nipple discharge, bleeding, retraction, ulceration, palpable mass, or skin thickening. (3) Tumor size: the diameter of the maximum cross-sectional area of the tumor was measured by ultrasound preoperatively, and the maximum diameter of the tumor in the gross pathologic samples was measured postoperatively. (4) Tumor location was categorized into central/retro-areolar and peripheral tumors according to preoperative ultrasound. Central tumors were those within the margin of the areola, while peripheral tumors were located outside of the areolar margin. (5) The shortest distance between the tumor and the nipple was measured as the tumor–nipple distance (TND) during microscopic examination of the tissue samples when available. (6) Tumor multicentricity/multifocality was defined as more than one lesion of invasive carcinoma separated by benign tissue. (7) Tumor type, including invasive ductal carcinoma (IDC), invasive lobular carcinoma (ILC), and ductal carcinoma *in situ* (DCIS). (8) Histology grade (Bloom–Richardson system). (9) Lymph node status. (10) ER, PR status (≥1% were positive, <1% were negative). (11) HER2 expression (immunohistochemistry or fluorescence *in situ* hybridization) status. (12) Ki-67 status.

Pathologic examination was performed on the vertical section of the nipple, and the sections were then analyzed using hematoxylin–eosin (HE) staining and immunohistochemical staining if necessary. The identification of tumor cells in the nipple sections was defined as nipple involvement, and we deemed the nipple to be involved if the nipple had invasive cancer, ductal carcinoma *in situ*, lobular carcinoma *in situ*, or Paget's disease.

### Statistical analysis

Student's *t*-test was used to evaluate continuous variables, and the chi-square test or Fisher's exact test was used to evaluate categorical variables. Only *P* < 0.05 was considered statistically significant. The relationship between the tumor ultrasound size and the histopathologic size was analyzed by using the Pearson correlation coefficient. Statistical calculations were performed by using SPSS software (version 26.0). Multivariate logistic regression analysis was performed to form a predictive model of NI on R software package (V 4.0.3).

## Results

### Patients' characteristics

A total of 250 patients were included in this study: 200 patients were included as the training group, and 50 patients were included as the validation group. The total NI rate was 12.6% (34/250), and patients with NI accounted for 14.5% (29/200) and 10.0% (5/50) in the training group and validation group, respectively. The details of the clinicopathologic characteristics of the training and validation groups are given in Table 1.

**Table 1:** Clinicopathologic characteristics of patients in the training and validation groups.

Characteristic	Training group				Validation group			
	Total	Negative for NI	Positive for NI	*P*-value	Total	Negative for NI	Positive for NI	*P*-value
Age	200			0.739	50			1.000
≤50	84	71 (84.5%)	13 (15.5%)		22	20 (90.9%)	2 (9.1%)	
> 50	116	100 (86.2%)	16 (13.8%)		28	25 (89.3%)	3(10.7%)	
Nipple signs	200			0.011	50			0.008
Normal	189	165 (87.3%)	24 (12.7%)		48	45 (93.8%)	3 (6.3%)	
Abnormal	11	6 (54.5%)	5 (45.5%)		2	0 (0.0%)	2 (100%)	
Tumor size (US)	200			0.001	50			0.138
≤4 cm	177	157 (88.7%)	20 (11.3%)		43	40 (93.0%)	3 (7.0%)	
> 4 cm	23	14 (60.9%)	9 (39.1%)		7	5 (71.4%)	2 (28.6%)	
Tumor size (P)	189			0.002	47			0.000
≤4 cm	166	147 (88.6%)	19 (11.4%)		39	39 (100.0%)	0 (0.0%)	
>4 cm	23	14 (60.9%)	9 (39.1%)		8	4 (50.0%)	4 (50.0%)	
TND(P)	48			0.005	14			0.143
≤1 cm	10	4 (40.0%)	6 (60.0%)		2	1 (50.0%)	1 (50.0%)	
> 1 cm	38	33 (86.8%)	5 (13.2%)		12	12 (100.0%)	0 (0.0%)	
Tumor location	200			0.000	50			0.005
Peripheral	169	154 (91.1%)	15 (8.9%)		45	43 (95.6%)	2 (4.4%)	
Central	31	17 (54.8%)	14 (45.2%)		5	2 (40.0%)	3 (60.0%)	
Multicentric/multifocal	200			0.112	50			0.486
Yes	34	26 (76.5%)	8 (23.5%)		7	7 (100.0%)	0 (0.0%)	
No	166	145 (87.3%)	21 (12.7%)		43	38 (88.4%)	5 (11.6%)	
Tumor type	193			0.301	49			0.359
DCIS	15	14 (93.3%)	1 (6.7%)		3	2 (66.7%)	1 (33.3%)	
IDC	164	140 (85.4%)	24 (14.6%)		45	41 (91.1%)	4 (8.9%)	
ILC	14	10 (71.4%)	4 (28.6%)		1	1 (100%)	0 (0.0%)	
Histology grade (IDC)	164			0.727	42			0.159
I	8	6 (75.0%)	2 (25.0%)		3	3 (100%)	0 (0.0%)	
II	101	87 (86.1%)	14 (13.9%)		21	17 (81.0%)	4 (19.0%)	
III	55	47 (85.5%)	8 (14.5%)		18	18 (100.0%)	0 (0.0%)	
Lymph node metastasis	199			0.002	48			1.000
Negative	120	110 (91.7%)	10 (8.3%)		25	23 (92.0%)	2 (8.0%)	
Positive	79	61 (77.2%)	18 (22.8%)		23	21 (91.3%)	2 (8.7%)	
HER2	183			0.025	44			1.000
Negative	117	106 (90.6%)	11 (9.4%)		26	25 (96.2%)	1 (3.8%)	
Positive	65	52 (78.8%)	14 (21.2%)		18	17 (94.4%)	1 (5.6%)	
ER	199			0.365	50			0.637
Negative	55	49 (89.1%)	6 (10.9%)		19	18 (94.7%)	1 (5.3%)	
Positive	144	121 (84.0%)	23 (16.0%)		31	27 (87.1%)	4 (12.9%)	
PR	199			0.648	50			0.383
Negative	55	48 (87.3%)	7 (12.7%)		21	20 (95.2%)	1 (4.8%)	
Positive	144	122 (84.7%)	22 (15.3%)		29	25 (86.2%)	4 (13.8%)	
Ki-67	199			0.833	50			0.301
< 15%	52	44 (84.6%)	8 (15.4%%)		10	9 (81.8%)	2 (18.2%)	
≥15%	148	127 (85.8%)	21 (14.2%)		40	36 (92.3%)	3 (7.7%)	

US, ultrasound; P, pathology; TND, tumor–nipple distance; IDC, invasive ductal carcinoma; ILC, invasive lobular carcinoma; ductal carcinoma in situ; ER, estrogen receptor; PR, progesterone receptor; HER2, HER2/neu amplification.

### The association between nipple involvement and clinicopathologic parameters

To explore the predictive potential of clinicopathologic parameters for NI, we analyzed the correlation between NI and clinicopathologic parameters. As seen in Table 1, in the training and validation groups, NI had no statistical correlation with patient age, multicentric/multifocal tumors, tumor type, histologic grade, estrogen receptor expression, progesterone receptor expression, or Ki-67 status.

In the training group, there was a significant difference in the lymph node metastasis (*P* = 0.002) and HER2 overexpression (*P* = 0.025) between patients with and those without NI. However, the difference was not statistically significant in the validation group.

Meanwhile, we observed that ultrasound tumor size, pathological tumor size, and TND all influenced the occurrence of NI in the training group. Patients with larger ultrasound tumor size had higher incidences of NI (>4 cm, 39.1% vs. ≤4 cm, 11.3%, *P* = 0.001). Consistently, patients with larger pathological tumor pathologic size had higher incidences of NI (>4 cm, 39.1% vs. ≤4 cm, 11.4%, *P* = 0.002). Meanwhile, patients with TND >1 cm and ≤1 cm had incidences of NI of 13.2% and 60.0%, respectively (*P* = 0.005). But no statistical difference was found with respect to tumor size measured by ultrasound and TND in the validation group.

Nevertheless, we found statistically significant differences between patients without and with NI in both groups when the tumor size was tested as numerical data. The median tumor size measured by ultrasound was 2.24 cm vs. 3.17 cm (*P* < 0.001) in the training group and the size was 2.49 cm vs. 3.92 cm (*P* = 0.006) in the validation group.

Both in the training and in the validation groups, there were significant differences in nipple signs (*P* = 0.011 and *P* = 0.008, respectively) and tumor location (*P* < 0.001 and *P* = 0.005, respectively) between patients with NI and those without NI. The presence of abnormal nipple signs increased vulnerability to NI. In the training group, the rates of NI of patients with abnormal nipple signs and patients with normal nipple signs were 45.5% and 12.7%, respectively (*P* = 0.011). Patients with tumor in the central location had a higher incidence of NI than those with tumors in the peripheral location (45.2% and 8.9%, respectively, *P* < 0.001).

Furthermore, the correlation between ultrasound tumor size and pathological tumor size was confirmed by the Pearson correlation coefficient (*r* = 0.608, *P* < 0.001), indicating that tumor size determined by ultrasound can accurately reflect the actual tumor histopathologic size.

### The predictive model for nipple involvement

**Figure 1:**
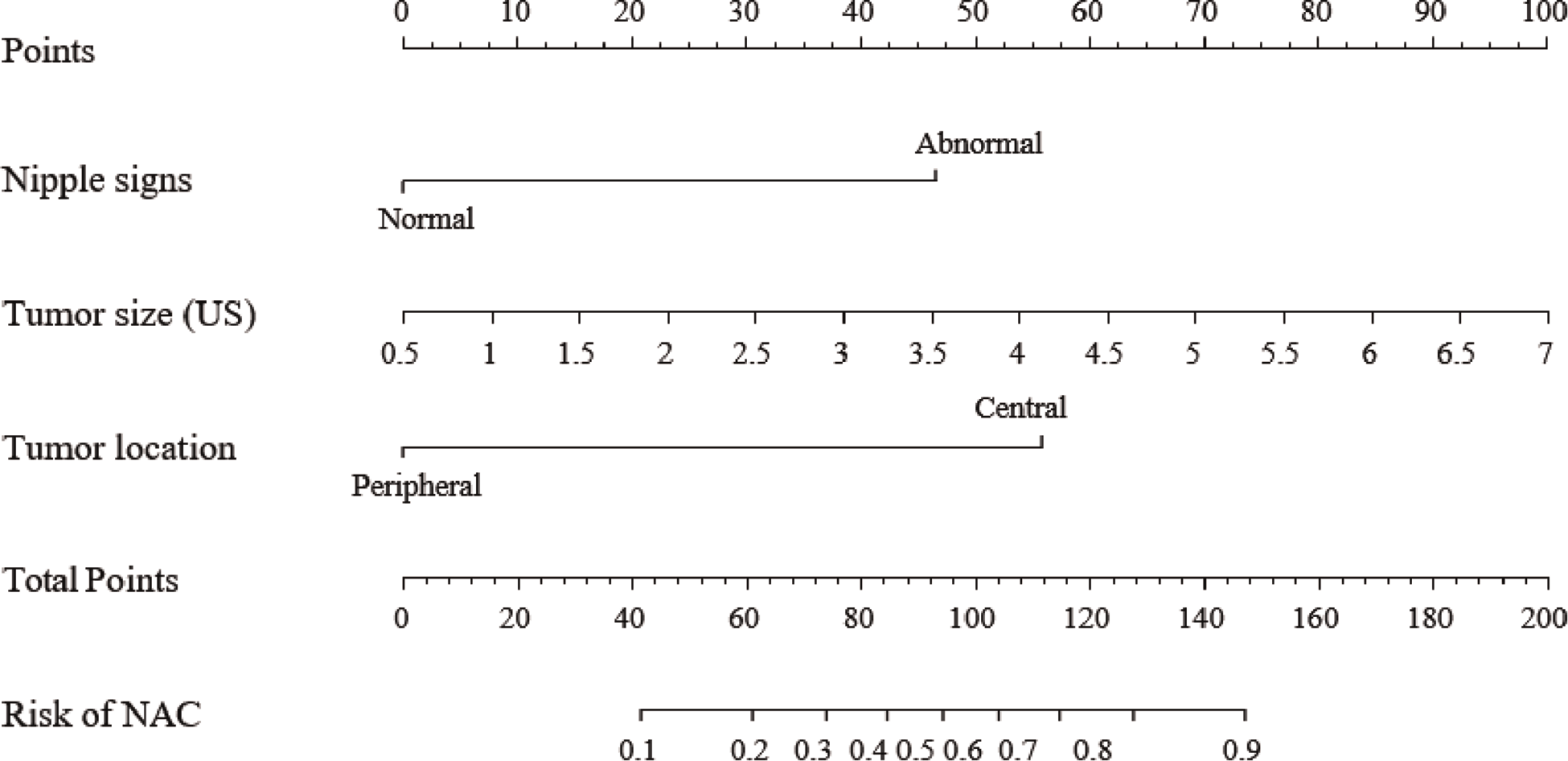
A clinical model nomogram for the prediction of NI. US, ultrasound; NI, nipple involvement.

Among the above clinicopathological factors associated with NI on univariable analysis, we selected three predictive factors that could be obtained preoperatively by physical examination and ultrasound imaging, namely, nipple signs (normal or abnormal), tumor size, and tumor location (central or peripheral). The multivariable regression analysis results in the training group are given in Table 2. The variance inflation factor (VIF) was 1.05–1.09, indicating that there was no co-linearity between the variances. The three parameters were used to develop a predictive model as a nomogram (Figure 1).

**Table 2:** Results of multivariate logistic regression models.

		Nomogram	AUC	Sensitivity	Specificity	Accuracy
Variables	Coefficient	OR (95% CI)	*P-*value			
Nipple signs	−1.55651	0.21(0.05−0.98)	0.048			
Tumor size (US)	1.94421	0.11(0.04−0.29)	<0.001			
Tumor location	−2.22006	6.99(2.18−22.43)	0.001			
Training group			0.86 (0.79−0.93)	86.2% (25/29)	70.7% (121/171)	73% (146/200)
Validation group			0.98 (0.95−1)	80% (4/5)	86.7% (39/45)	86% (43/50)

US, ultrasound; OR, odds ratio; CI, confidence interval; AUC, area under the curve.

**Figure 2:**
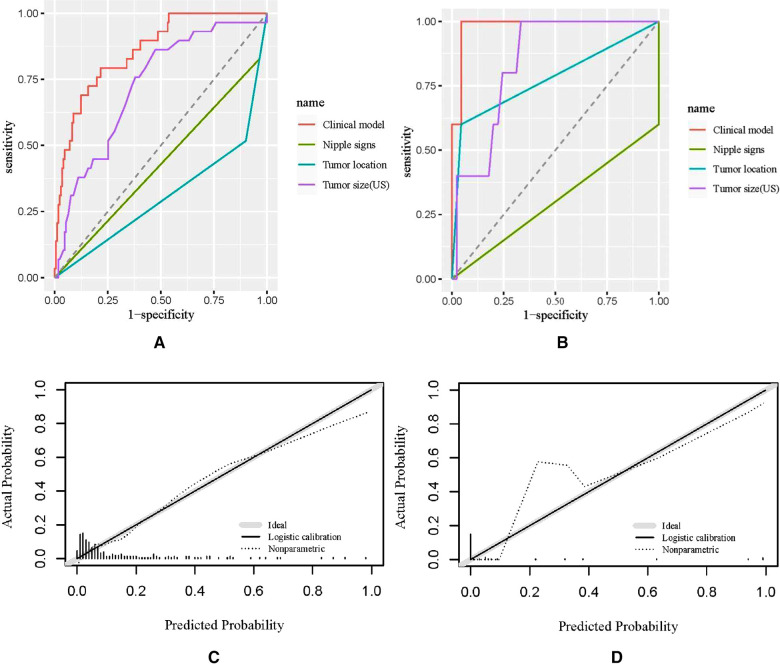
Receiver operating characteristic (ROC) curves and calibration curves. (**A,B**) ROC curves of the training group and the validation group; (**C,D**) calibration curves of the nomogram in the training group and the validation group. US, ultrasound.

**Figure 3:**
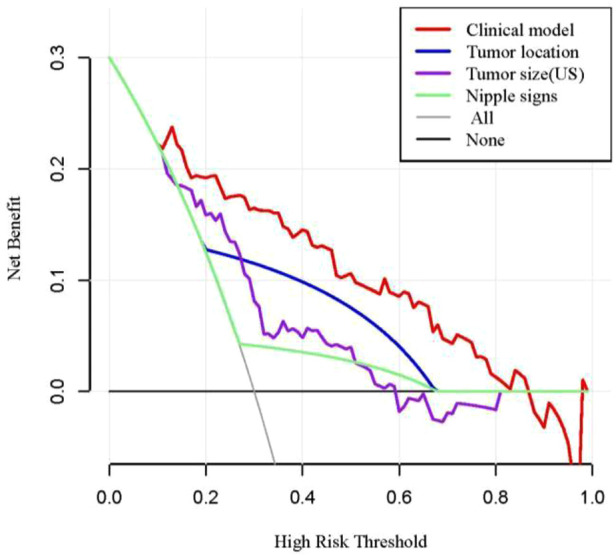
DCA of the clinical model in predicting NI. DCA, decision curve analysis; US, ultrasound.

Receiver operating characteristic (ROC) analysis was applied to the clinical model (Figure 2A,B), the area under the curve (AUC) in the training group and validation group was 0.858 (95% CI, 0.79–0.92) and 0.982 (95% CI, 0.94–1.00), respectively, and calibration curves (Figure 2C,D) showed good agreement between prediction and observation in both groups. The Hosmer–Lemeshow test showed a *P*-value of 0.585 in the training group and 0.89 in the validation group, suggesting that our clinical model was a good fit. The three variables were incorporated as clinical predictive models: nipple signs [OR: 5.88 (1.61–20.0); *P*=0.007], ultrasound tumor size [OR: 6.94 (2.32–20.8); *P* =0.001], and tumor location [OR: 8.33 (3.45–20.0); *P* < 0.001]. In the training group, the clinical model had a specificity of 70.7%, a sensitivity of 86.2%, and an accuracy of 73.0%. In the validation group, the clinical model had a specificity of 86.7%, a sensitivity of 80.0%, and an accuracy of 86.0%. As presented in Figure 3, the decision curve analysis (DCA) demonstrated that the clinical model had maximum application values when the threshold probability ranged from 0.2 to 0.8.

## Discussion

As the emphasis today is on aesthetic outcomes and quality of life after treatment for breast cancer, NSM is being increasingly performed for patients undergoing mastectomy with reconstruction (4–6). As reported, the rates of nipple involvement in breast cancer range from 5.6% to 58% (15–18, 22–26). Also, local cancer recurrence rates (1.7%–10.3%) after NSM have been reported in published studies (27–30). Hence, it is important to appropriately select patients for the oncological safety of NSM. In this study, we developed a clinical model nomogram for NSM patient selection based on the abnormal nipple signs, ultrasound reported tumor size, and tumor location.

First, we observed that patients with abnormal nipple signs (nipple discharge, bleeding, retraction, ulceration, palpable mass, and thickened skin) were more likely to have NI both in the training and in the validation groups. Billar et al. found that abnormal nipple signs or symptoms had a 61% sensitivity, 86% specificity, 45% positive predictive value (PPV), and 92% negative predictive value (NPV) for determining NI (27).

Although nipple discharge is one of the most common symptoms of breast cancer, it is not a contraindication for nipple preservation if there is no evidence of tumor invasion to the nipple margin (28). Nipple discharge is not necessarily the outcome of tumor invasion of the nipple, which only acts as a drain channel when breast cancer invades ducts located far from the center (29). Therefore, an evaluation of other factors is indispensable.

In our research, all patients underwent ultrasound imaging preoperatively, which described and recorded the tumor location and tumor size. Both in the training and in the validation groups, we found that patients with tumor in the central location had a higher incidence of NI than those with tumors in the peripheral location. Banerjee et al. also observed only 4 (2.5%) of 160 patients with tumors located in the peripheral location, compared with 40 (68%) of 59 patients with tumors located in the central or retro-areolar areas of the breast (*P* < 0.001) (30). Tumor size has been found to be a significant predictive factor of NI (31, 32). We noticed that tumor size measured by ultrasound was associated with NI in the training group (*P* = 0.001) when it was dichotomized into ≤4 cm or >4 cm, but in the validation group, the *P*-value was 0.138. However, the sample volume in the validation group was small, and therefore, the true connection may not be proved. Indeed, we found that tumor size was associated with NI in the training group (*P* < 0.001) and validation group (*P* = 0.006) when it was tested as numerical data. In addition, Pearson correlation coefficient analysis demonstrated that preoperative ultrasound can accurately represent histopathologic tumor size. Hence, we confirmed the functional role of preoperative ultrasound imaging and recommend its application during the management of NSM.

As reported, pathological tumor size, TND, lymph node status, and HER2 overexpression showed a significant correlation with NI (31, 33–36), which was also demonstrated in the training group of our study, but the association was not proved in our validation group. The roles of these factors in predicting NI deserve further exploration. Because these characteristics are known only after mastectomy, we did not include them in the final predictive model.

Relevant to the predictive model, a recent study produced a preoperative predictive model using seven factors, namely, MRI tumor size ≥4 cm, mammographic TND <1 cm, MRI TND <1 cm, MRI nipple enhancement, central tumor, multicentric/multifocal involvement, and clinical node involvement. Each factor had a score of 0 or 1, and the total scores were used to categorize patients into low (0–3), intermediate (4), or high (5–7) risk groups. It was recommended that the nipple should be be sacrificed in patients in the high-risk group and that patients in the intermediate-risk group who hoped to preserve the nipple should be undergo frozen section examination (14). Another study by Wang et al. proposed a model consisting of tumor location, nuclear grade, and HER2 expression (34). Schecter et al. reported a formula for predicting NI based on tumor size, TND, and stage, which was found to have a sensitivity of 92% and a specificity of 77% (37). The models developed in these studies were based on high-cost imaging or preoperative biopsy. Factors such as pathological TND, tumor size, number of metastatic lymph nodes, and HER2 overexpression were generally known only after surgery. However, the parameters of the predictive model in our study were much easier to obtain. Based on ultrasound tumor size, tumor location, and nipple signs, a predictive model was proposed to predict the possibility of nipple involvement.

There are several limitations in our retrospective study. First, our model was implemented in a single center with a relatively low number of patients. Second, the ultrasound imaging of patients was performed by doctors of different backgrounds and with varied experiences, which might result in selection bias. Third, there was a lack of information on the TND of most patients owing to inadequate information on this aspect.

## Conclusion

In our study, the rate of incidence of NI was 12.6% in mastectomy patients, and the associated clinicopathologic characteristics included nipple signs, tumor location, tumor size measured by ultrasound or gross pathologic samples, tumor–nipple distance, lymph node metastasis, and HER2 overexpression. We developed an effective predictive model as a nomogram based on nipple signs, tumor size measured by ultrasound, and tumor location that helped improve the accuracy of selecting eligible patients for NSM.

## Data Availability

The raw data supporting the conclusions of this article will be made available by the authors, without undue reservation.
